# Nonmotor Symptoms in *LRRK2* G2019S Associated Parkinson’s Disease

**DOI:** 10.1371/journal.pone.0108982

**Published:** 2014-10-17

**Authors:** Carles Gaig, Dolores Vilas, Jon Infante, María Sierra, Inés García-Gorostiaga, Mariateresa Buongiorno, Mario Ezquerra, Maria José Martí, Francesc Valldeoriola, Miquel Aguilar, Matilde Calopa, Jorge Hernandez-Vara, Eduardo Tolosa

**Affiliations:** 1 Parkinson’s Disease and Movement Disorders Unit, Neurology Service, Institut de Neurociències Hospital Clínic, University of Barcelona, Barcelona, Spain; 2 Institut d’investigacions Biomèdiques August Pi i Sunyer (IDIBAPS), Barcelona, Spain; 3 Centro de Investigación Biomédica en Red de Enfermedades Neurodegenerativas (CIBERNED), Madrid, Spain; 4 Neurology Service, Hospital Universitario Marqués de Valdecilla, University of Cantabria (UC), Santander, Spain; 5 Neurology Service, Hospital de Galdakao, Usansolo, Vizcaya, Spain; 6 Neurology Service, Hospital Universitari Mutua de Terrasa, Barcelona, Spain; 7 Neurology Service, Hospital Universitari de Bellvitge, Barcelona, Spain; 8 Neurology Service, Hospital Universitari Vall D’Hebron, Barcelona, Spain; Philadelphia VA Medical Center, United States of America

## Abstract

**Background:**

Idiopathic Parkinson’s disease (IPD) and *LRRK2*-associated PD (*LRRK2*-PD) might be expected to differ clinically since the neuropathological substrate of *LRRK2*-PD is heterogeneous. The range and severity of extra-nigral nonmotor features associated with *LRRK2* mutations is also not well-defined.

**Objective:**

To evaluate the prevalence and time of onset of nonmotor symptoms (NMS) in *LRRK2*-PD patients.

**Methods:**

The presence of hyposmia and of neuropsychiatric, dysautonomic and sleep disturbances was assessed in 33 *LRRK2*-G2019S-PD patients by standardized questionnaires and validated scales. Thirty-three IPD patients, matched for age, gender, duration of parkinsonism and disease severity and 33 healthy subjects were also evaluated.

**Results:**

University of Pennsylvania Smell Identification Test (UPSIT) scores in *LRRK2*-G2019S-PD were higher than those in IPD (23.5±6.8 vs 18.4±6.0; p = 0.002), and hyposmia was less frequent in G2019S carriers than in IPD (39.4% vs 75.8%; p = 0.01). UPSIT scores were significantly higher in females than in males in *LRRK2*-PD patients (26.9±4.7 vs 19.4±6.8; p<0.01). The frequency of sleep and neuropsychiatric disturbances and of dysautonomic symptoms in *LRRK2*-G2019S-PD was not significantly different from that in IPD. Hyposmia, depression, constipation and excessive daytime sleepiness, were reported to occur before the onset of classical motor symptoms in more than 40% of *LRRK2*-PD patients in whom these symptoms were present at the time of examination.

**Conclusion:**

Neuropsychiatric, dysautonomic and sleep disturbances occur as frequently in patients with *LRRK2*-G2019S-PD as in IPD but smell loss was less frequent in LRRK2-PD. Like in IPD, disturbances such as hyposmia, depression, constipation and excessive daytime sleepiness may antedate the onset of classical motor symptoms in *LRRK2*-G2019S-PD.

## Introduction

Mutations in the leucine-rich repeat kinase 2 gene *(LRRK2)* are the most common cause of inherited parkinsonism and account for a significant proportion of familial and sporadic Parkinson’s disease (PD) cases [1]–[2]. The neuropathological substrate in *LRRK2*-PD is in some cases quite different from idiopathic PD (IPD) and ranges from brainstem or diffuse Lewy body pathology, to nigral degeneration without distinctive histopathology, and to progressive supranuclear palsy–like pathology. Age at disease onset and parkinsonian motor features are similar between *LRRK2* related PD (LRRK2-PD) and idiopathic PD (IPD) [3] but studies assessing nonmotor symptoms (NMS) in *LRRK2*-PD patients are limited and results at times conflicting [4]–[6]. Dysautonomia, sleep and mood disturbances as well as other common NMS occurring in IPD result, in part, from extra-nigral lesions in the brain and the peripheral autonomic nervous system [7]. The heterogeneous neuropathology of *LRRK2*-PD suggests that the range and severity of extra-nigral NMS could differ from those encountered in IPD.

The aim of the present study was to evaluate the prevalence and estimate the onset of NMS in patients with *LRRK2* G2019S PD.

## Methods

### Subjects

A sample of 1251 PD patients from two regions of Spain, Catalonia (Hospital Clínic de Barcelona, Hospital Mútua de Terrassa, Hospital Vall d’Hebron and Hospital de Bellvitge) and Cantabria (Hospital Marqués de Valdecilla, Santander) was screened for LRRK2 G2019S and codon 1441 (R1441G/C/H) mutations as previously described [3]. PD was diagnosed according to UK Parkinson’s Disease Society criteria [8]. LRRK2 G2019S carriers were proposed to participate in this study. LRRK2-PD patients that met diagnostic criteria for dementia in PD (PDD) [9] were excluded, to avoid interference of cognitive impairment in the evaluation of NMS. A group of 33 IPD patients were recruited as controls among those from the initial sample of 1180 patients that tested negative for LRRK2 mutations. We selected prospectively on a case by case basis those patients who matched to the LRRK2 subjects for age, gender, duration of parkinsonism (from onset of motor symptoms, OMS) and disease severity (Hoehn and Yahr (H-Y) stage), agreed to participate in the study, did not fulfil criteria for PDD [9], and had not family history for PD. We also studied age and gender matched healthy subjects (HS) without PD, dementia, any other neurological disorder or a positive family history for PD. Patients, their relatives as well as medical staff of participating centre recruited volunteers from their personal relations who were screened for exclusion criteria and were enrolled as controls for this study. The study was approved by the Hospital Clinic of Barcelona ethics committee and the written informed consent was obtained from all study subjects.

### Parkinsonism evaluation

Information about motor symptoms (rest tremor, bradykinesia, rigidity, postural instability and persistent asymmetry), as well as development of levodopa-induced motor complications, freezing of gait and repeated falls during the disease course, was collected by means of a structured clinical interview. All patients were assessed through the Unified Parkinson’s Disease Rating Scale (UPDRS), Schwab & England scale and H-Y stage in On condition. Medications at the time of the evaluation were also recorded and levodopa equivalent daily dose (LEDD) calculated [10].

### Nonmotor symptoms evaluation

The presence of the following NMS was assessed: hyposmia; *neuropsychiatric disorders:* depression, anxiety, hallucinations and psychosis; *dysautonomic symptoms:* constipation and urinary dysfunction; and *sleep disturbances*: REM Sleep Behaviour Disorder (RBD), excessive daytime sleepiness (EDS) and insomnia. A structured clinical interview was conducted to determine the presence of each NMS at the time of the evaluation. When a NMS was present, we asked the patient to estimate when it had been developed in relation to the OMS (e.g. before, concurrently or after). Each NMS was also evaluated by using several self-administered tests. Information on the current use of medications, such as laxatives, hypnotics or antidepressants to treat some of these NMS, was also collected.

*Smell loss* was assessed by asking to the subjects whether they experienced a loss or change in their ability to smell. The 40-items University of Pennsylvania Smell Identification Test (UPSIT; Smell Identification Test™ Sensonics, Spanish version) [11] was administered. Factors that could impair odor identification, such as active smoking habit, past-history of significant traumatic head injury or rhinologic disorders, were also considered. UPSIT scores obtained previously from 148 HS aged 30 to 85 year-old were used for comparison with *LRRK2*-PD and IPD. Since odor identification has been shown to decline with age and is better in females than in males [11]–[12], these 148 HS were stratified by age (<50 years, n = 36; 50–69 years, n = 76; and ≥70, n = 36) and gender (males, n = 73; females, n = 75). Mean and standard deviation (SD) UPSIT score for each age-gender HS subgroup was calculated. Hyposmia was considered to be present if the UPSIT score was lower than the mean–2SD corresponding to the age-gender matched HS subgroup.

*Presence of depression and anxiety* at the time of this study were diagnosed according to DSM-IV criteria [13]. In addition, a previous history of depression or anxiety was also recorded. The Hospital Anxiety and Depression scale (HADS) [14] was used to quantify the symptoms. To assess the presence of hallucinations and psychosis the NINS-NIMH work group criteria for psychosis in PD [15] and the Parkinsonian Psychosis Rating Scale (PPRS) were used [16].

*Constipation* was diagnosed according to the Rome criteria [17]. Bowel movements frequency was evaluated. The Bristol Scale Stool was used to assess whole gut transit time [18]. *Urinary dysfunction* was assessed by asking for long-lasting complaints of urinary urgency, frequency, incontinence or incomplete emptying. The SCOPA-AUT was administered [19].

RBD was considered to be present when a history of problematic sleep behaviors that were potentially harmful, disrupted sleep continuity or was annoying to self or bed partner was present [20]. EDS occurred when the subject could fall asleep at least twice a day and the total sleeping time during the daytime was more than 1 hour [21] and *insomnia* when there were long-lasting complaints of difficulty in initiating or maintaining sleep. The Pittsburgh sleep quality index (PSQI), the Epworth sleepiness scale (ESS) and the Parkinson’s disease sleep scale (PDSS) were administered [22]–[24].

#### Statistical analyses

Categorical variables and the frequency of each NMS in the different groups were assessed by using the Chi-square test or the Fisher’s exact test when appropriated. For continuous variables, the Kruskal-Wallis analysis and the Mann-Whitney *U* test were used to compare the means of groups for multiple comparisons and in pairs, respectively. To determine whether there was a relationship between each NMS and other variables, the Spearman correlation coefficient was obtained. A significance level of <0.05 was used. P-values were also calculated using the false discovery rate (FDR) correction for multiple comparisons. The statistical analyses were performed using commercially available software (SPSS, Version18.0).

## Results

### General demographic data and parkinsonian motor symptoms

Among 66 PD patients identified as *LRRK2* G2019S carriers, only 33 were included in the study (22 from Barcelona and 11 from Santander). One *LRRK2*-PD patient was excluded because of severe dementia. Of the remaining 32 non-participants, 10 declined to participate, 11 had died when the study was initiated and 11 had been lost for follow up. Thirty-three IPD patients and 33 HS (22 from Barcelona and 11 from Santander in each group) were included. Mean age was not statistically different between *LRRK2*-PD, IPD and HS (Table 1). Family history for PD was positive in 20 (60.6%) of *LRRK2*-PD patients. There were no significant differences in the features of motor symptoms, or in dopamine replacement treatment (Table 1).

**Table 1:** General demographic data and parkinsonian motor symptoms in patients with *LRRK2* G2019S associated Parkinson’s disease, idiopathic Parkinson’s disease and healthy subjects.

	*LRRK2* PD (n = 33)	IPD (n = 33)	HS (n = 33)	P
**Age** (years)^1^	64.8±11.4	65.1±10.0	64.8±10.2	**0.99** ¶
**Sex** (male, %)	15 (45.4%)	15 (45.4%)	15 (45.4%)	**1.0** #
**Disease duration** (years)^1^	9.2±5.7 (2–28)	9.0±6.1 (1–30)		**0.79** §
**Motor symptoms during disease course; n (%)**				
Rest tremor	29 (87.9%)	27 (81.8%)		**0.49** #
Action tremor	20 (60.6%)	23 (69.7%)		**0.44** #
Bradykinesia	33 (100%)	33 (100%)		**1.0** ±
Postural instability	16 (48.5%)	11 (33.3%)		**0.21** #
Repeated falls	9 (27.3%)	7 (21.2%)		**0.57** #
Freezing of gait	18 (54.6%)	14 (42.4%)		**0.32** #
Persistent Asymmetry	31 (93.9%)	31 (93.9%)		**1.0** ±
Fluctuations	18 (54.5%)	17 (51.5%)		**0.80** #
Dyskinesias	19 (57.6%)	16 (48.5%)		**0.46** #
**UPDRS part II** 1	10.3±7.5 (0–26)	8.3±4.9 (0–24)		**0.48** §
**UPDRS part III** 1	24.0±14.0 (0–62)	19.9±12.2 (2–62)		**0.16** §
**UPDRS part IVA+IVB** 1	3.21±3.7 (0–13)	2.0±2.7 (0–10)		**0.18** §
**Hoehn & Yahr stage** 1	2.1±1.0 (1–4)	1.8±0.8 (1–4)		**0.24** §
**Schwab & England** 1	82.1±14.7 (50–100)	85.8±10.3 (60–100)		**0.44** §
**Treatment with levodopa; n (%)**	31 (93.9%)	30 (90.9%)		**1.0** ±
**Treatment with dopamine agonists; n (%)**	27 (81.8%)	25 (75.8%)		**0.55** #
**Levodopa equivalent daily dose** (in mg)^1^	793.7±482.1 (105–2744)	823.0±516.4 (0–1770)		**0.77** §

*LRRK2* G2019S PD: *LRRK2* G2019S related Parkinson’s disease; IPD: Idiopathic Parkinson’s disease; HS: healthy subjects.

1 Mean ± Standard deviation (Range).

¶ Kruskal-Wallis analysis;

§ Mann-Whitney U test;

# Chi-square test;

± Fisher’s exact test.

### Nonmotor symptoms

#### Olfaction

**Figure 1:**
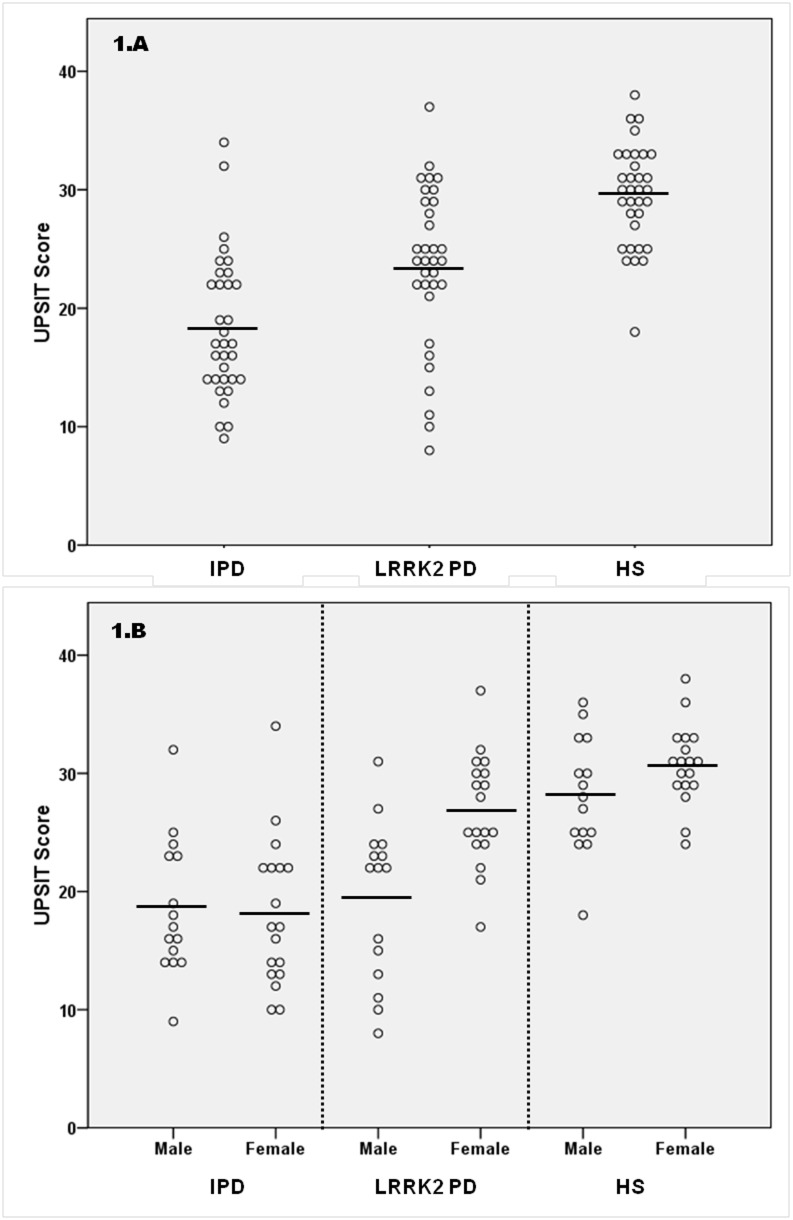
University of Pennsylvania Smell Identification Test (UPSIT) scores. UPSIT scores in LRRK2 G2019S Parkinson’s disease patients, idiopathic Parkinson’s disease patients and healthy controls (Figure 1.A). UPSIT score in each group separated by sex (Figure 1.B). Circles represent individual values, while the bar refers to the mean UPSIT score in each group. IPD: idiopathic Parkinson’s disease; LRRK2-PD: LRRK2 associated Parkinson’s disease, HS: healthy subjects.

Awareness of smell loss occurred in a similar frequency in both *LRRK2*-PD and IPD patients (54.5% vs 63.6%; p = 0.45; Table 2) and more frequently than in HS. Mean UPSIT scores, though, were significantly different among the three groups. *LRRK2*-PD patients had a mean UPSIT score significantly higher than IPD patients (23.5±6.8 vs 18.4±6.0; p = 0.002) and lower than HS (29.5±4.3; p = 0.001) (Figure 1. A). Hyposmia was present in 13 (39.4%) of G2019S carriers, significantly lower than in IPD (25 (75.8%); p = 0.01) (Table 2). No significant differences were found in the frequency of active smoking, rhinologic pathology or history of head trauma between groups.

**Table 2:** Nonmotor symptoms in patients with *LRRK2* G2019S associated Parkinson’s disease, idiopathic Parkinson’s disease and healthy subjects.

	*LRRK2*-PD (n = 33)	IPD (n = 33)	Controls (n = 33)	Three groups comparison	FDR p	*LRRK2* vs IPD	*LRRK2* vs controls	IPD vs controls
**Change in smell sense**	18 (54.5%)	21 (63.6%)	5 (15.2%)	0.021* #	0.055	**0.45** #	0.02* #	0.007* #
**UPSIT Score** 1	23.5±6.8	18.4±6.0	29.5±4.3	0.001¶	0.007	**0.002** §	0.001§	0.001§
**Hyposmia** 2	13 (39.4%)	25 (75.8%)	1(3.0%)	0.001* #	0.007	**0.01** #	0.003* #	0.001* #
**History of depression**	19 (57.6%)	12 (36.4%)	4 (12.1%)	0.024* #	0.055	**0.08** #	0.006* #	0.02* #
**Active depression**	6/19 (31.5%)	8/12 (66.6%)	1/4 (25.0%)	0.445#	0.703			
**Antidepressive treatment**	5/19 (26.3%)	9/12 (75.0%)	1/4 (25.0%)	0.24#	0.45			
**History of anxiety**	8 (24.2%)	9 (27.3%)	5 (15.2%)	0.609#	0.794			
**Current active anxiety**	2/8 (25.0%)	6/9 (66.7%)	3/5 (60.0%)	0.559#	0.772			
**Treatment for anxiety**	3/8 (37.5%)	2/9 (22.2%)	3/5 (60.0%)	0.642#	0.802			
**HADS total**	10.4±5.5	10.1±6.6	8.0±5.6	0.26¶	0.459			
*HADS depression*	5.2±2.7	5.0±3.9	3.0±2.9	0.001* ¶	0.007	**0.54** §	0.001* §	0.02* §
*HADS anxiety*	4.9±3.6	5.1±3.8	5.0±3.5	0.98¶	0.985			
**Hallucinations**	3 (9%)	5 (15%)	NA			**0.40** ±		
**PPRS score** 1	0.3±0.5	0.5±0.9	0.03±17	0.018* ¶	0.055	**0.54** §	0.012* §	0.001* §
**Constipation**	10 (30.3%)	13 (39.4%)	9 (27.3%)	0.74#	0.853			
**Bowel movement** (week)^1^	6.5±2.7	5.6±1.7	6.5±2.0	0.13¶	0.278			
**Bristol scale** 1	3.5±1.1	2.9±0.9	3.8±1.1	0.002* ¶	0.012	**0.03** * §	0.28§	0.001* §
**Urinary dysfunction**	17 (51.5%)	15 (45.5%)	8 (24.2%)	0.283#	0.472			
**SCOPA-AUT** 1	16.4±8.7	13.5±8.5	10.5±5.8	0.02* ¶	0.055	**0.17** §	0.001* §	0.14§
*G-I*	4.3±3.4	4.1±3.5	2.1±2.3	0.024* ¶	0.055	**0.83** §	0.16§	0.02* §
*Urinary*	6.4±4.8	5.4±3.8	4.9±3.2	0.56¶	0.077			
**RBD symptoms**	7 (21.2%)	14 (42.4%)	1 (3.0%)	0.008* #	0.03	**0.11** #	0.054#	0.01* #
**Insomnia**	22 (66.7%)	19 (57.6%)	20 (60.6%)	0.932#	0.985			
**EDS**	6 (18.2%)	13 (39.4%)	0	0.003* #	0.013	**0.057** #	0.02* #	0.001* #
**Global PSQI** 1	7.3±5.4	6.7±4.8	5.2±4.7	0.16¶	0.32			
**Total PDSS** 1	116.4±16.8	116.7±22.3	130.1±3.4	0.001* ¶	0.007	**0.72** §	0.001* §	0.02* §
**ESS Score** 1	8.1±4.6	9.1±5.3	5.6±2.8	0.003* ¶	0.013	**0.46** §	0.02* §	0.001* §

*LRRK2* G2019S PD: *LRRK2* G2019S-related Parkinson’s disease; IPD: Idiopathic Parkinson’s disease; HS: healthy subjects. G-I: Gastro-intestinal; RBD: REM behavior disorder; EDS: excessive daytime sleepiness.

# Chi-square test;

± Fisher’s exact test;

¶ Kruskal-Wallis analysis;

§ Mann-Whitney *U* test;

*Statistically significant: *P<*0.05;

1 Mean ± Standard deviation (Range);

2 Hyposmia was arbitrarily defined as an UPSIT score lower than the mean–2 SD UPSIT score obtained in a subset of healthy subjects of the same gender and similar age.

Mean UPSIT score in *LRRK2*-PD cases was significantly higher in females than in males (26.9±4.7 vs 19.4±6.8, p<0.01; Figure 1.B). Hyposmia was present in only 3 (16.7%) LRRK2-PD females, in contrast to 10 (66.7%) *LRRK2*-PD males. Such significant difference in UPSIT scores by gender was not observed in IPD patients (18.6±5.8 males vs 18.2±6.3 females; p = 0.66) or HS (28.1±4.9 males vs 30.7±3.4 females; p = 0.10). *LRRK2*-PD females were significantly older than *LRRK2*-PD males (67.8±8.9 vs 61.1±13.3; p = 0.04), but disease duration or severity, and frequency of active smoking, rhinologic pathology or history of head trauma, were similar in both genders. Mean UPSIT score in *LRRK2*-PD females was significantly higher than in IPD females (p<0.01) and significantly lower than in HS females (p = 0.01). In contrast, mean UPSIT score in *LRRK2*-PD males was similar to IPD males (p = 0.71), and significantly lower than in HS males (p<0.01). No correlation was found between UPSIT score and disease duration or severity in *LRRK2*-PD or IPD patients.

#### Neuropsychiatric symptoms

Current active depression and current treatment with antidepressive drugs were more frequent in IPD than in *LRRK2*-PD, but these differences were not statistically significant (Table 2). There were no differences in the frequency of *anxiety disorder* between *LRRK2*-PD, IPD and HS. Nine IPD patients were under antidepressive treatment but only 8 had active depression. Similarly, 3 *LRRK2*-PD patients were under anxiety treatment but only 2 had active anxiety. These patients were treated with antidepressants and benzodiazepines because of past history of depression and anxiety despite improvement of symptoms. The HADS subscore for depression was similar between *LRRK2-*PD and IPD, but higher than HS. The HADS subscore for anxiety was similar between groups (Table 2). *Hallucinations* occurred in similar frequency in *LRRK2*-PD and IPD. In all *LRRK2* and IPD patients, hallucinations were visual in nature. Delusions or severe psychosis did not occur in any *LRRK2*-PD nor IPD patient.

#### Dysautonomic symptoms

No significant differences were found in the presence of *constipation* between *LRRK2*-PD, IPD and HS groups (Table 2). Mean Bristol scale stool score was significantly higher in *LRRK2*-PD than in IPD (Table 2). Mean SCOPA-AUT subscore for gastro-intestinal dysfunction was similar in *LRRK2*-PD and IPD. IPD patients, but not *LRRK2*-PD patients, showed mean SCOPA-AUT subscore for gastro-intestinal dysfunction significantly higher compared to HS. The presence of *urinary dysfunction* was not statistically different between three groups. No differences in mean SCOPA-AUT subscore for urinary dysfunction was identified among the three groups (Table 2).

#### Sleep disturbances

A trend to an increased frequency of RBD symptoms in IPD compared to *LRRK2*-PD was found. Frequency of insomnia and use of hypnotics were similar among the three groups. IPD patients had a borderline increased frequency of EDS compared to *LRRK2*-PD (Table 2). Mean Global PSQI, total PDSS and EDSS scores were not significantly different between *LRRK2*-PD and IPD. *LRRK2*-PD and IPD patients had a mean Global PSQI score that was not significantly different from HS. In contrast, *LRRK2*-PD and IPD patients showed a mean total PDSS significantly lower than HS. Mean ESS score in *LRRK2* and IPD were significantly higher compared to HS (Table 2).

### Estimated onset of NMS in *LRRK2*-PD

*LRRK2*-PD patients frequently reported that several NMS occurred before OMS (table 3). Smell loss, depression, constipation and EDS were reported to develop before OMS in more than 40% of the *LRRK2*-PD patients in whom these symptoms were present at the time of examination. Smell loss and depression were reported to occur at variable time intervals before OMS but constipation and EDS frequently were estimated to occur more than 10 years before OMS in most *LRRK2*-PD (Table 3). In *LRRK2*-PD subjects, RBD was reported to occur usually coincidentally or after OMS. In IPD patients smell loss, depression and constipation were also reported to appear before OMS, but EDS and RBD were reported to appear coincidentally or after OMS in most cases. Anxiety symptoms were reported coincidentally or developing after OMS in most *LRRK2*-PD cases but more frequently before OMS in IPD.

**Table 3:** Estimated presence of nonmotor symptoms in *LRRK2* G2019S Parkinson’s disease patients and idiopathic Parkinson’s disease patients in relation to onset of motor symptoms.

	*LRRK2*-PD	IPD
**Loss or change in smell sense; n**	**18**	**21**
***Coincidentally or after OMS (n;%)***	**8 (44.4%)**	**8 (38.1%)**
***Before OMS (n;%)***	**8 (44.4%)**	**8 (38.1%)**
More than 10 yrs before OMS	2/8 (25%)	4/8 (50%)
Within 10 yrs before OMS	4/8 (50%)	2/8 (25%)
Within 2 yrs before OMS	2/8 (25%)	2/8 (25%)
Unknown	2 (11.1%)	5 (23.8%)
**Depression; n**	**19**	**12**
***Coincidentally or after OMS (n;%)***	**11 (57.9%)**	**7 (58.3%)**
***Before OMS (n;%)***	**8 (42.1%)**	**5 (41.7%)**
More than 10 yrs before OMS	2/8 (25%)	2/5 (40%)
Within 10 yrs before OMS	3/8 (37.5%)	2/5 (40%)
Within 2 yrs before OMS	3/8 (37.5%)	1/5 (20%)
**Anxiety symptoms; n**	**8**	**9**
***Coincidentally or after OMS (n;%)***	**6 (75%)**	**1 (11.1%)**
***Before OMS (n;%)***	**2 (25%)**	**8 (88.9%)**
More than 10 yrs before OMS	0	5/8 (62.5%)
Within 10 yrs before OMS	0	3/8 (37.5%)
Within 2 yrs before OMS	2/2 (100%)	0
**Constipation; n**	**14**	**15**
***Coincidentally or after OMS (n;%)***	**5 (35.7%)**	**8 (53.3%)**
***Before OMS (n;%)***	**9 (64.3%)**	**7 (46.7%)**
More than 10 yrs before OMS	8/9 (88.9%)	7/7 (100%)
Within 10 yrs before OMS	1/9 (11.1%)	0
Within 2 yrs before OMS	0	0
**Urinary dysfunction; n**	**17**	**15**
***Coincidentally or after OMS (n;%)***	**12 (70.6%)**	**12 (80%)**
***Before OMS (n;%)***	**5 (29.4%)**	**3 (20%)**
More than 10 yrs before OMS	0	0
Within 10 yrs before OMS	1/5 (20%)	1/3 (33.3%)
Within 2 yrs before OMS	4/5 (80%)	2/3 (66.6%)
**RBD symptoms; n**	**7**	**14**
***Coincidentally or after OMS (n;%)***	**5 (71.4%)**	**12 (85.7%)**
***Before OMS (n;%)***	**2 (28.6%)**	**2 (14.3%)**
More than 10 yrs before OMS	0	0
Within 10 yrs before OMS	½ (50%)	0/2 (0.0%)
Within 2 yrs before OMS	½ (50%)	2/2 (100%)
**Insomnia; n**	**19**	**22**
***Coincidentally or after OMS (n;%)***	**16 (84.2%)**	**14 (63.6%)**
***Before OMS (n;%)***	**3 (15.8%)**	**8 (36.4%)**
More than 10 yrs before OMS	2/3 (66.6%)	6/8 (75%)
Within 10 yrs before OMS	0	0
Within 2 yrs before OMS	1/3 (33.3%)	2/8 (25%)
**EDS; n**	**6**	**13**
***Coincidentally or after OMS (n;%)***	**3 (50%)**	**12 (92.3%)**
***Before OMS (n;%)***	**3 (50%)**	**1 (7.7%)**
More than 10 yrs before OMS	2/3 (66.6%)	1/1 (100%)
Within 10 yrs before OMS	1/3 (33.3%)	0
Within 2 yrs before OMS	0	0

*LRRK2* G2019S PD: *LRRK2* G2019S related Parkinson’s disease; IPD: Idiopathic Parkinson’s disease, OMS: onset of motor symptoms, RBD: REM sleep behavior disorder, EDS: excessive daytime sleepiness.

## Discussion

Our study shows that NMS occur frequently in *LRRK2* G2019S PD patients, in a frequency similar to a group of IPD subjects of similar disease duration, severity of motor symptoms, and dopaminergic treatment. The only NMS that significantly differed between *LRRK2*-PD and IPD was smell loss. While reported awareness of smell loss occurred as frequently in *LRRK2*-PD as in IPD, UPSIT scores were significantly higher in *LRRK2*-PD. Hyposmia was present in only 39% of our *LRRK2*-PD patients, in contrast to 75% of IPD patients. Also in other studies the prevalence of abnormal olfaction in *LRRK2*-PD has been found to range from 36 to 49%, significantly inferior to IPD (75–81%) [5].

Reasons for the differences in smell between *LRRK2*-PD and IPD encountered in this and other studies remain unclear. Heterogeneous pathology or less severe involvement of olfactory structures in *LRRK2*-PD has been proposed to explain such differences. In Parkin gene associated PD, absence of smell loss is common [25] and neuropathological changes are usually limited to the substantia nigra without LB pathology [26]. One study involving four *LRRK2-PD* brains, reported α-synuclein accumulation in the olfactory bulb, olfactory tract, and primary olfactory cortex providing a pathophysiologic substrate for olfactory deficit^6^. Still only 20% of *LRRK2* G2019S cases are thought to present different pathology to that seen in IPD [27], and therefore neuropathological heterogeneity may not adequately explain the preservation of the olfactory sense in most *LRRK2* patients.

In contrast to previous studies, we found that smell was particularly preserved in G2019S *LRRK2*-PD females. Such difference could suggest a gender effect in the expression of *LRRK2*-PD. Gender-related susceptibility factors likely play a role in PD, a disorder more common in males [28] and female predominance has been suggested to occur in G2019S *LRRK2*-PD [29]. One study has also reported an earlier age at disease onset in women than in men with the G2019S mutation [4].

Neuropsychiatric disorders, dysautonomic symptoms and sleep disturbances were frequent in *LRRK2* G2019S carriers, similarly to that observed in IPD. Depression, anxiety and hallucinations were present in our *LRRK2* patients at a frequency similar to that reported in the literature [4], [30], [31]. Sleep disturbances like RBD, insomnia and EDS are common in PD and have been reported to be present frequently in patients carrying the G2019S mutation. The frequency of RBD symptoms tended to be lower in *LRRK2*-PD cases than in IPD, although the difference was not statistically significant. The prevalence of RBD in IPD reported in the literature varies from 15% to 46% [32]. A recent study has reported RBD symptoms in only 11% of the *LRRK2*-PD patients, compared to 42% of IPD patients [33], suggesting that RBD could be less frequently present in *LRRK2*-PD. Frequency of constipation and urinary dysfunction in our *LRRK2* patients was similar to that reported by others [5], [31], [33]. In our study the frequency of constipation was similar between *LRRK2* and IPD, but mean Bristol scale stool score was significantly lower in *LRRK2*-PD than in IPD, suggesting that intestinal motility could be less impaired in *LRRK2*-PD.

A substantial proportion of our *LRRK2*-PD patients reported that several NMS, such as hyposmia, depression, constipation, or EDS, had been present before OMS. Some NMS have been evaluated in a limited number of asymptomatic subjects carrying the *LRRK2* G2019S mutation [6], [31], [34] and hyposmia, depression and constipation have been found to occur in some non-manifesting carriers [31], [34], although some of these premotor features were found to be no more frequent in non-manifesting carriers than in non-carriers. The presence of these symptoms in the premotor phase, as in the case of IPD, suggests that the neuropathological changes occur probably in non-dopaminergic brainstem and peripheral nervous system structures before the involvement of the substantia nigra, as suggested by Braak [35].

Some limitations of our study should be mentioned. Some results are based on patient’s responses. More information could be obtained with objective tests, as occurred in smell evaluation, where no differences were identified when asked for a loss or change in smell, while the UPSIT was able to detect significant differences. Also since the mean disease duration was 9 years, we acknowledge that the patient’s responses in regards to symptoms occurring in the presymptomatic phase may not be accurate,but others studies have reported NMS such as these occurring in the premotor phase of LRRK2-PD [6], [30]. Future prospective studies in asymptomatic LRRK2 G2019S carriers should clarify this issue. Finally, cognitive dysfunction which is among the most relevant and disabling NMS in PD, was not assessed in detail in our study, but only one patient was excluded for the study because of dementia. Some studies have suggested that the frequency of dementia could be lower than usually reported in IPD [5], [36]. Cognitive dysfunction in LRRK2 patients deserves additional and specifically designed studies. One strength of our study, in contrast to previous ones that usually assessed one or only a few NMS, include the evaluation of several NMS in the same group of patients which has given a more global impression of the contribution of NMS to the clinical picture of LRRK2 associated PD. In addition, our study includes similar NMS assessments in a group of healthy subjects for comparison purposes.

In summary, neuropsychiatric, dysautonomic and sleep disturbances are equally frequent in non-demented *LRRK2* G2019S PD and IPD. Olfactory dysfunction, however, occurs less often in *LRRK2* G2019S PD, maybe reflecting less involvement of olfactory structures by the neurodegenerative process. Smell function seems to be particularly preserved in females with the G2019S mutation, suggesting a gender effect in the expression of some *LRRK2*-PD symptoms. Some NMS may antedate the onset of PD motor syndrome in substantial number of patients carrying *LRRK2* G2019S mutation, indicating that a premotor phase similar to that occurring in IPD probably occurs in this genetic form of PD, and that common physiopathological mechanism probably underlie the onset and progression of the disease in both *LRRK2* G2019S PD and IPD.

## References

[1] Paisán Ruíz C, Jain S, Evans EW, Gilks WP, Simón J, et al (2004) Cloning of the gene containing mutations that cause PARK8-linked Parkinson’s disease. Neuron 44(4): 595–600. 15541308 10.1016/j.neuron.2004.10.023

[2] Gilks WP, Abou Sleiman PM, Gandhi S, Jain S, Singleton A, et al (2005) A common LRRK2 mutation in idiopathic Parkinson’s disease. Lancet 365: 415–6. 15680457 10.1016/S0140-6736(05)17830-1

[3] Gaig C, Ezquerra M, Marti MJ, Munoz E, Valldeoriola F, et al (2006) LRRK2 mutations in Spanish patients with Parkinson disease: frequency, clinical features, and incomplete penetrance. Arch Neurol 63: 377–82. 16533964 10.1001/archneur.63.3.377

[4] Goldwurm S, Zini M, Di Fonzo A, De Gaspari D, Siri C, et al (2006) LRRK2 G2019S mutation and Parkinson’s disease: a clinical, neuropsychological and neuropsychiatric study in a large Italian sample. Parkinsonism Relat Disord 12: 410–9. 16750929 10.1016/j.parkreldis.2006.04.001

[5] Healy DG, Falchi M, O’Sullivan SS, Bonifati V, Durr A, et al (2008) Phenotype, genotype, and worldwide genetic penetrance of LRRK2-associated Parkinson’s disease: a case-control study. Lancet Neurol 7: 583–90. 18539534 10.1016/S1474-4422(08)70117-0 PMC2832754

[6] Silveira Moriyama L, Guedes LC, Kingsbury A, Ayling H, Shaw K, et al (2008) Hyposmia in G2019S LRRK2-related parkinsonism: clinical and pathologic data. Neurology 71: 1021–6. 18809839 10.1212/01.wnl.0000326575.20829.45

[7] Simuni T, Sethi K (2008) Nonmotor manifestations of Parkinson’s disease. Ann Neurol 64: S65–80. 19127582 10.1002/ana.21472

[8] Hughes AJ, Daniel SE, Kilford L, Lees AJ (1992) Accuracy of clinical diagnosis of idiopathic Parkinson’s disease: a clinico-pathological study of 100 cases. J Neurol Neurosurg Psychiatry 55: 181–4. 1564476 10.1136/jnnp.55.3.181 PMC1014720

[9] Emre M, Aarsland D, Brown R, Burn DJ, Duyckaerts C, et al (2007) Clinical diagnostic criteria for dementia associated with Parkinson’s disease. Mov Disord 22: 1689–707. 17542011 10.1002/mds.21507

[10] Möller JC, Körner Y, Dodel RC, Meindorfner C, Stiasny Kolster K, et al (2005) Pharmacotherapy of Parkinson’s disease in Germany. J Neurol 252: 926–35. 15765268 10.1007/s00415-005-0784-1

[11] Doty RL, Bromley SM, Stern MB (1995) Olfactory testing as an aid in the diagnosis of Parkinson’s disease: development of optimal discrimination criteria. Neurodegeneration 4: 93–7. 7600189 10.1006/neur.1995.0011

[12] Doty RL, Shaman P, Applebaum SL, Giberson R, Siksorski L, et al (1984) Smell identification ability: changes with age. Science 226: 1441–3. 6505700 10.1126/science.6505700

[13] Washington (1994) American Psychiatric Association. Diagnostic and statistical manual of mental disorders: DSM-IV.

[14] Zigmond AS, Snaith RP (1983) The hospital anxiety and depression scale. Acta Psychiatr Scand 67: 361–70. 6880820 10.1111/j.1600-0447.1983.tb09716.x

[15] Ravina B, Marder K, Fernandez HH, Friedman JH, McDonald W, et al (2007) Diagnostic criteria for psychosis in Parkinson’s disease: report of an NINDS, NIMH work group. Mov Disord 22: 1061–8. 17266092 10.1002/mds.21382

[16] Friedberg G, Zoldan J, Weizman A, Melamed E (1998) Parkinson Psychosis Rating Scale: a practical instrument for grading psychosis in Parkinson’s disease. Clin Neuropharmacol 21: 280–4. 9789707

[17] Longstreth GF, Thompson WG, Chey WD, Houghton LA, Mearin F, et al (2006) Functional bowel disorders. Gastroenterology 130: 1480–91. 16678561 10.1053/j.gastro.2005.11.061

[18] Riegler G, Esposito I (2001) Bristol scale stool form. A still valid help in medical practice and clinical research. Tech Coloproctol 5: 163–4. 11875684 10.1007/s101510100019

[19] Visser M, Marinus J, Stiggelbout AM, Van Hilten JJ (2004) Assessment of autonomic dysfunction in Parkinson’s disease: the SCOPA-AUT. Mov Disord 19: 1306–12. 15390007 10.1002/mds.20153

[20] Medicine AASM (2005) International Classification of Sleep Disorders- Second Edition.

[21] Gjerstad MD, Aarsland D, Larsen JP (2002) Development of daytime somnolence over time in Parkinson’s disease. Neurology 58: 1544–6. 12034797 10.1212/wnl.58.10.1544

[22] Buysse DJ, Reynolds CF, Monk TH, Berman SR, Kupfer DJ (1989) The Pittsburgh Sleep Quality Index: a new instrument for psychiatric practice and research. Psychiatry Res 28: 193–213. 2748771 10.1016/0165-1781(89)90047-4

[23] Johns MW (1991) A new method for measuring daytime sleepiness: the Epworth sleepiness scale. Sleep 14: 540–5. 1798888 10.1093/sleep/14.6.540

[24] Chaudhuri KR, Pal S, DiMarco A, Whately Smith C, Bridgman K, et al (2002) The Parkinson’s disease sleep scale: a new instrument for assessing sleep and nocturnal disability in Parkinson’s disease. J Neurol Neurosurg Psychiatry 73: 629–35. 12438461 10.1136/jnnp.73.6.629 PMC1757333

[25] Khan NL, Katzenschlager R, Watt H, Bhatia KP, Wood NW, et al (2004) Olfaction differentiates parkin disease from early-onset parkinsonism and Parkinson disease. Neurology 62: 1224–6. 15079034 10.1212/01.wnl.0000118281.66802.81

[26] Hayashi S, Wakabayashi K, Ishikawa A, Nagai H, Saito M, et al (2000) An autopsy case of autosomal-recessive juvenile parkinsonism with a homozygous exon 4 deletion in the parkin gene. Mov Disord 15: 884–8. 11009195 10.1002/1531-8257(200009)15:5<884::aid-mds1019>3.0.co;2-8

[27] Poulopoulos M, Levy OA, Alcalay RN (2012) The neuropathology of genetic Parkinson’s disease. Mov Disord 27: 831–42. 22451330 10.1002/mds.24962 PMC3383342

[28] Wooten GF, Currie LJ, Bovbjerg VE, Lee JK, Patrie J (2004) Are men at greater risk for Parkinson’s disease than women? J Neurol Neurosurg Psychiatry 75: 637–9. 15026515 10.1136/jnnp.2003.020982 PMC1739032

[29] Orr Urtreger A, Shifrin C, Rozovski U, Rosner S, Bercovich D, et al (2007) The LRRK2 G2019S mutation in Ashkenazi Jews with Parkinson disease: is there a gender effect? Neurology 69: 1595–602. 17938369 10.1212/01.wnl.0000277637.33328.d8

[30] Kasten M, Kertelge L, Brüggemann N, van der Vegt J, Schmidt A, et al (2010) Nonmotor symptoms in genetic Parkinson disease. Arch Neurol 67: 670–6. 20558386 10.1001/archneurol.67.6.670

[31] Marras C, Schüle B, Munhoz RP, Rogaeva E, Langston JW, et al (2011) Phenotype in parkinsonian and nonparkinsonian LRRK2 G2019S mutation carriers. Neurology 77: 325–33. 21753163 10.1212/WNL.0b013e318227042d PMC3140802

[32] Iranzo A, Santamaria J, Tolosa E (2009) The clinical and pathophysiological relevance of REM sleep behavior disorder in neurodegenerative diseases. Sleep Med Rev 13: 385–401. 19362028 10.1016/j.smrv.2008.11.003

[33] Ruiz Martínez J, Gorostidi A, Goyenechea E, Alzualde A, Poza JJ, et al (2011) Olfactory deficits and cardiac 123I–MIBG in Parkinson’s disease related to the LRRK2 R1441G and G2019S mutations. Mov Disord 26: 2026–31. 21611983 10.1002/mds.23773

[34] Saunders Pullman R, Stanley K, Wang C, San Luciano M, Shanker V, et al (2011) Olfactory dysfunction in LRRK2 G2019S mutation carriers. Neurology 77: 319–24. 21753159 10.1212/WNL.0b013e318227041c PMC3140803

[35] Braak H, Del Tredici K, Rub U, de Vos RA, Jansen Steur EN, et al (2003) Staging of brain pathology related to sporadic Parkinson’s disease. Neurobiol Aging 24: 197–211. 12498954 10.1016/s0197-4580(02)00065-9

[36] Brockmann K, Gröger A, Di Santo A, Liepelt I, Schulte C, et al (2011) Clinical and brain imaging characteristics in leucine-rich repeat kinase 2-associated PD and asymptomatic mutation carriers. Mov Disord 26: 2335–42. 21989859 10.1002/mds.23991

